# *Candida albicans* and *Staphylococcus* Species: A Threatening Twosome

**DOI:** 10.3389/fmicb.2019.02162

**Published:** 2019-09-18

**Authors:** Hans Carolus, Katrien Van Dyck, Patrick Van Dijck

**Affiliations:** ^1^Laboratory of Molecular Cell Biology, Department of Biology, Institute of Botany and Microbiology, KU Leuven, Leuven, Belgium; ^2^VIB-KU Leuven Center for Microbiology, Leuven, Belgium

**Keywords:** *Candida albicans*, *Staphylococcus aureus*, *Staphylococcus epidermidis*, polymicrobial infections, biofilms, interspecies interactions, interkingdom interactions

## Abstract

*Candida albicans* and *Staphylococcus* species are, respectively, the most common fungal and bacterial agents isolated from bloodstream infections, worldwide. Moreover, it has been shown that 20% of all *C. albicans* bloodstream infections are polymicrobial in nature, with *Staphylococcus epidermidis* and *Staphylococcus aureus* being the first and third most common co-isolated organisms, respectively. These species are part of the commensal microbial flora but can cause hospital-acquired infections with an extreme ability to inhabit diverse host niches, especially in immunocompromised patients. They are well known for their ability to form persistent biofilms in the host or on abiotic surfaces such as indwelling medical devices. Interactions within these biofilm communities can lead to increased virulence, drug tolerance, and immune evasion. This can ultimately impact morbidity and infection outcome, often leading to an increased mortality. Therefore, characterizing the interactions between these species could lead to the development of novel therapeutic approaches that target polymicrobial infections. In this mini review, we briefly highlight the current knowledge and most recent insights into the complex interspecies interactions of *C. albicans* with *Staphylococcus* bacteria.

## Introduction

*Candida albicans* is a commensal fungus that colonizes the oral cavity, vagina, and gastro-intestinal tract in most humans (Nobile and Johnson, 2015). It is however also an opportunistic pathogen, able to cause both superficial and systemic infections, the latter mainly in immunocompromised patients. In certain niches of the host, *C. albicans* co-exists with commensal bacteria, including *Staphylococcus* species (Tsui et al., 2016). *Staphylococcus epidermidis* and *Staphylococcus aureus* are, respectively, the first and third most co-isolated pathogens with *C. albicans* in systemic blood stream infections. Additionally, more than one in four cases of candidemia is estimated to be polymicrobial, whereas systemic *Staphylococcus* infections without *Candida* are less common (Klotz et al., 2007a).

The significance of polymicrobial infections caused by a mixture of bacteria and fungi is increasingly recognized in medical settings (Brogden et al., 2005). Furthermore, a vast number of infections, including those of *C. albicans* and *Staphylococcus* species, originate from biofilms and they are often associated with high mortality rates (Tsui et al., 2016). *C. albicans* and *Staphylococcus* species were co-isolated from various biofilm-associated diseases, including periodontitis, cystic fibrosis, denture stomatitis, urinary tract, burn wound infections, and infections of medical devices such as central venous catheters (Adam et al., 2002; Baena-Monroy et al., 2005; Gupta et al., 2005; Valenza et al., 2008; Cuesta et al., 2010). The complexity of these polymicrobial infections poses an additional challenge to find efficient treatment strategies (Koo et al., 2017).

This mini review elaborates on the current knowledge and recent insights in the interactions, altered drug tolerance and infection outcome, and possible treatment strategies of polymicrobial *C. albicans*-*Staphylococcus* species infections.

## Molecular Interactions of *C. Albicans* With *Staphylococcus* Species

### Physical Interactions

Different research groups have focused on the characterization of the interaction between *C. albicans* and *Staphylococcus* species at a molecular level. An early study by El-Azizi and colleagues showed enhanced adhesion and biofilm formation of *C. albicans* upon addition of *S. epidermidis*, indicating a possible symbiotic interaction between both species (El-Azizi et al., 2004). Peters et al. observed that staphylococci, with *S. aureus* in particular, predominantly adhere to the hyphae of *C. albicans* resulting in a unique biofilm architecture (Peters et al., 2010).

**Figure 1:**
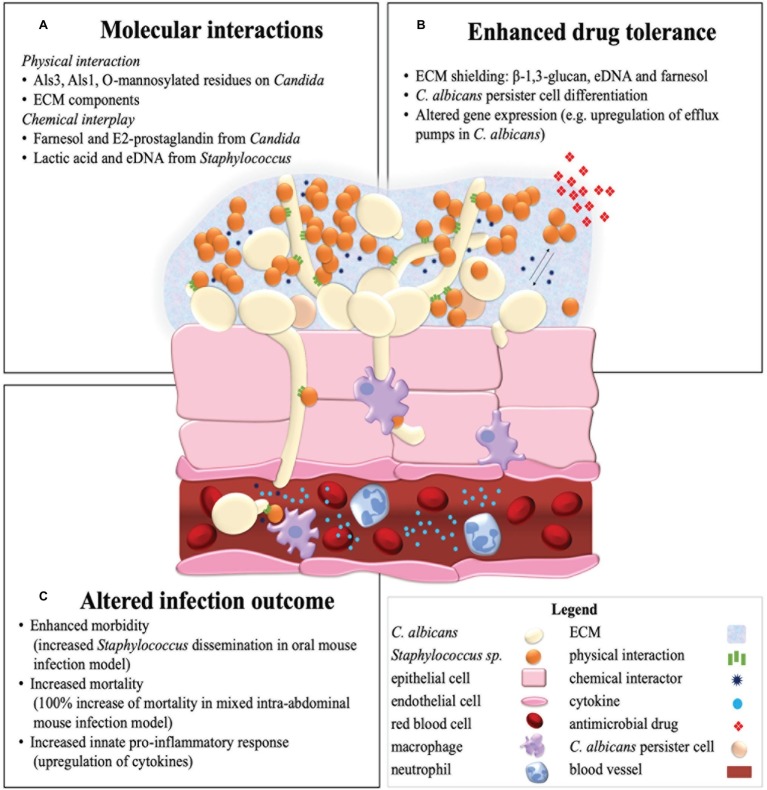
Summary illustration of a mixed *C. albicans* and *Staphylococcus* species biofilm in a host environment. Represented are the molecular interactions of *C. albicans* and *Staphylococcus* species **(A)** in an invasive endothelial infection with an enhanced antimicrobial drug tolerance **(B)** and altered infection outcome **(C)** as a result.

Later, the importance of *C. albicans* Als membrane proteins in *C. albicans*-*Staphylococcus* species aggregation was recognized (Klotz et al., 2007b). While Als5, Als6, and Als7 adhesins are associated with adhesion to human endothelium, the Als3 protein was identified as a *C. albicans* hyphae specific receptor which binds *S. aureus* (Peters et al., 2012) and *S. epidermidis* (Beaussart et al., 2013). These hyphae specific associations of *S. aureus* (Ovchinnikova et al., 2012) and *S. epidermidis* (Beaussart et al., 2013) further stresses the importance of *C. albicans* yeast-to-hyphae morphogenesis in mixed species biofilm formation. However, it was observed that even in the *C. albicans als3* mutant strain, *S. aureus* cells attach to the fungal hyphae, suggesting that either additional cell surface molecules, biofilm extracellular matrix (ECM) components or non-specific hydrophobic and electrostatic interactions may play a role in interspecies aggregation as well (Peters et al., 2012). A single-cell force spectroscopy study suggested that *C. albicans* Als1 and cell surface *O*-mannosylations interact with specific peptide ligands containing an “τφ+” motif and lectin binding sites on the *S. epidermidis* surface, respectively (Beaussart et al., 2013). Specific and direct interspecies cell surface interactions between *C. albicans* and *Staphylococcus* species might at least partly explain the enhanced biofilm growth (El-Azizi et al., 2004), drug tolerance (Adam et al., 2002; Harriott and Noverr, 2010; Kong et al., 2016), and increased morbidity (Kong et al., 2015; Nash et al., 2016) in polymicrobial infections (Figure 1A).

### Chemical Interplay

In one of the earliest studies of *C. albicans*-*S. aureus* interactions, it was suggested that secreted products of *C. albicans* may be the cause of the enhanced growth of *S. aureus* in co-infection (Staib et al., 1976). Besides physical interactions, secreted effectors such as quorum sensing (QS) molecules and small secreted metabolites indeed prove to be involved in interspecies communication. Quorum sensing, i.e., density dependent intercellular signaling through small secreted molecules, has been found in both *Staphylococcus* (Kong et al., 2006) and *Candida* species (Albuquerque and Casadevall, 2012). During aggregation or biofilm formation, inter and intra-kingdom QS intensifies and might be able to act as a significant biofilm developmental regulator (Morales and Hogan, 2010). The most widely described QS molecule of *C. albicans* is farnesol (Hornby et al., 2001). Farnesol reduces *C. albicans* yeast-to-hyphae morphogenesis and so consequently reduces biofilm growth (Ramage et al., 2002; Piispanen et al., 2011). Moreover, it was found that farnesol reduced *S. aureus* viability and impedes *S. aureus* biofilm development when present in high concentrations (>100 μM) (Jabra-Rizk et al., 2006). On the contrary, it is shown that low concentrations of farnesol (0.5–5 nM) promote *S. aureus* biofilm formation (Krause et al., 2015). Moreover, intermediate concentrations of farnesol (40 μM) that are physiologically relevant within the *C. albicans* biofilm were found to enhance drug tolerance of *S. aureus* toward vancomycin (Kong et al., 2017). It was hypothesized that farnesol induces oxidative stress in *S. aureus*, which is sensed by the multiple gene regulator MgrA that in term upregulates drug efflux pumps in a general stress response (Kong et al., 2017). Recently, this effect of farnesol on *S. aureus* was studied in more detail. Vila et al. found that *S. aureus* that is repeatedly exposed to farnesol loses its typically yellow pigment staphyloxanthin, which is an important virulence factor. Moreover, farnesol exposure induced an accumulation of intracellular reactive oxygen species (ROS) and alterations in the expression of redox-sensors and major virulence regulators. Interestingly, this activated stress-response led to an enhanced tolerance to H_2_O_2_ and phagocytic killing (Vila et al., 2019). Finally, it was discovered that the increased host mortality of co-infections by *C. albicans* and *S. aureus* was associated with *C. albicans*-mediated induction of the *agr* QS system of *S. aureus*, with increased alpha and delta-toxin production and hemolytic activity as a result (Todd et al., 2019). It is likely that other, yet unidentified QS molecules may also play a stimulatory role in the interkingdom interaction of *Candida* and *Staphylococcus* species. This hypothesis is strengthened by the observation that conditioned medium of *S. aureus* biofilms dramatically increases *C. albicans* biofilm growth (Lin et al., 2013).

Besides quorum sensing molecules, other effectors such as secreted metabolites and macromolecules take part in *C. albicans*-*Staphylococcus* species interactions. Prostaglandin E2 (PGE_2_) is, in contrast to farnesol, a secreted metabolite of *C. albicans* that increases *S. aureus* biofilm growth in a dose-dependent manner (Krause et al., 2015). Similarly, extracellular DNA (eDNA) from *S. epidermidis* and to a lesser extent from *C. albicans*, which is released into the ECM by autolysis or active secretion, enhances biofilm adhesion, aggregation, and development (Pammi et al., 2013). Downregulation of the bacterial repressors of autolysis (the *lrg* operon) in the mixed *C. albicans*-*S. epidermidis* biofilm might be the cause of enhanced eDNA levels in this condition (Pammi et al., 2013). Moreover, lactate, which is a small secreted metabolite present in the human host but also produced by anaerobic fermentation in bacteria including staphylococci, can inhibit yeast-to-hyphae morphogenesis (Liang et al., 2016) and induce β-glucan masking (Ballou et al., 2016) in *C. albicans*, both strategies of immune evasion. To conclude, Xu et al. (2008) impose a role of bacterial peptidoglycans in the induction of *C. albicans* hyphae formation (Xu et al., 2008) and thus, potentially, biofilm development. Further research can potentially reveal the role of chemical interspecies communication in mixed species biofilms with the ultimate goal of impeding commensal or symbiotic molecular interactions within this polymicrobial relationship (Figure 1A).

## Enhanced Antimicrobial Drug Tolerance

A significant amount of evidence shows that single species biofilms of fungi and bacteria are characterized by increased drug tolerance levels, compared to their planktonic counterparts. This biofilm specific enhanced tolerance is mostly due to various biofilm specific traits, including ECM production, persister cell differentiation and upregulation of drug efflux pumps (Van Acker et al., 2014). Mixed *C. albicans***-***Staphylococcus* species biofilms display an additional, polymicrobial specific enhanced drug tolerance toward various antimicrobials, compared to their single species biofilm format (Adam et al., 2002; Harriott and Noverr, 2009, 2010; Kong et al., 2016; Kean et al., 2017). Multiple authors show enhanced tolerance of *S. aureus* toward the antibiotic vancomycin in the presence of *C. albicans* (Harriott and Noverr, 2009, 2010; Kong et al., 2016). This effect is mediated by *C. albicans* ECM (Harriott and Noverr, 2009; Kong et al., 2016) and by the transcriptional regulator of biofilm formation Bcr1, but independent from *C. albicans* Als1–7, Als9, and Hwp1 adhesins (Harriott and Noverr, 2010). Similarly, *C. albicans* ECM increases vancomycin tolerance of *S. epidermidis* in the mixed *C. albicans***-***S. epidermidis* biofilm (Adam et al., 2002). Vancomycin remains one of the few effective antibiotics against the clinically relevant methicillin-resistant *S. aureus* (MRSA), stressing the importance of this polymicrobial commensal effect (Kong et al., 2016). Also *C. albicans* can benefit from the polymicrobial interaction during treatment. Biofilms of *C. albicans* with *S. epidermidis* and *S. aureus* show enhanced tolerance toward the antifungals fluconazole (Adam et al., 2002) and miconazole (Kean et al., 2017), respectively.

Two mechanisms can possibly explain the protective effect of ECM in polymicrobial interactions: the matrix binds drug components and thus physically limiting penetration of drugs into the biofilm, or the matrix material itself induces drug tolerance associated gene expression (Adam et al., 2002; Harriott and Noverr, 2009). This first hypothesis is supported by Kong et al., showing the significant importance of *C. albicans* matrix β-1,3-glucan in polymicrobial tolerance toward vancomycin of *S. aureus* by decreasing penetration of the drug inside the biofilm (Kong et al., 2016). A similar role might exist for eDNA, another important ECM constituent of *C. albicans*-*Staphylocoocus* species biofilms (Pammi et al., 2013; Kean et al., 2017). Furthermore, it was shown that degradation of eDNA by DNAse treatment increased the sensitivity of miconazole by two-fold in the mixed *C. albicans*-*S. epidermidis* biofilm but not in mono-species biofilms, while ECM chitin and β-1,3-glucan degradation had no such effect (Kean et al., 2017). The polymicrobial biofilm produces significantly more matrix material. It is hypothesized that most of it is made by *C. albicans*, which than coats *S. aureus* (Harriott and Noverr, 2009). However, it is also possible that in a mixed culture, *S. aureus* upregulates its own ECM production. Besides the protection by polymeric ECM constituents, other mechanisms can play a role in the enhanced polymicrobial drug tolerance. An example of this is farnesol of *C. albicans,* which enhances drug tolerance of *S. aureus* toward vancomycin (Kong et al., 2017). Further research into the molecular mechanisms involved in the polymicrobial enhanced drug tolerance can possibly lead to improved treatment of polymicrobial *C. albicans*-*Staphylococcus* infections (Figure 1B).

## Altered Infection Outcome

### Enhanced Morbidity and Mortality

Co-infections of *C. albicans* with *Staphylococcus* species are among the best examples of “lethal synergism,” meaning that the co-infection mediates the increased mortality of otherwise “survivable” monomicrobial infections. Several studies by Carlson et al. show that peritoneal infections of *C. albicans* with *S. aureus* are lethal, while the monomicrobial infections are not (Carlson, 1982, 1983, 1988). Other studies using an intra-abdominal infection mouse model found an increased mortality rate of 80–100% at 48–72 h post-inoculation with *C. albicans* and *S. aureus*, while the single species infections were nonlethal (Nash et al., 2014, 2016; Esher et al., 2019; Todd et al., 2019). In addition, this co-infection resulted in both local and systemic inflammation and dissemination and a significant increase in the infection rates and microbial burden. Mice infected with *S. aureus* had on average 1 × 10^4^ CFU (colony forming units) in the kidney and spleen which increased with a median 1-log-unit or 2-log-unit, respectively, when co-infected with *C. albicans*. A 4-log-unit CFU increase was observed both in the spleen and kidneys for *C. albicans* in the mixed infections, compared to the single species infection (Peters and Noverr, 2013). In a murine model of oral candidiasis, a co-infection of *C. albicans* with *S. aureus* showed on average a 5-log-unit increase in *S. aureus* colonization on the tongues of mice which were co-infected with *C. albicans*, compared to mice infected with *S. aureus* alone (Kong et al., 2015). In addition, it was shown that oral candidiasis predisposes for secondary systemic bacterial infections since *S. aureus* was recovered from the kidneys of co-infected mice, but not from single species infections. *C. albicans* was recovered in 30% of the co-infected mice. Furthermore, these mice showed signs of severe morbidity with a mortality rate up to 20%, while the monomicrobial infections were nonlethal (Kong et al., 2015; Schlecht et al., 2015). The ability to form hyphae was found to be crucial in the murine model of oral candidiasis, in contrast to the intra-abdominal infection model, where *C. albicans* morphogenesis is not required to induce synergistic lethality (Nash et al., 2014). Since *C. albicans* forms hyphae which can invade and penetrate human tissues (Sudbery et al., 2004) and staphylococci adhere specifically to these hyphae (Peters et al., 2010), *C. albicans* hyphae possibly taxi staphylococci during invasion (Peters et al., 2012).

Enhanced mortality is also observed during co-infection of *C. albicans* with *S. epidermidis.* Clinical studies in neonates with concurrent bloodstream infections of *C. albicans* and coagulase-negative staphylococci, such as *S. epidermidis*, showed an increased mortality and morbidity compared to the single species infections (Kaufman et al., 2014). Additionally, Holt et al. used a polymicrobial biofilm model of *S. epidermidis* and *C. albicans* in the nematode *Caenorhabditis elegans* to show that *S. epidermidis* exhibits a significantly increased virulence and induced mortality in dual infections. Extracellular polymeric substances (EPSs) produced by *S. epidermidis* may play a role in this enhanced virulence (Holt et al., 2017). The infectious synergism of these species highlights the importance of both the treatment and surveillance of mixed species infections (Figure 1C).

### Effect on the Host Immune Response

Little is known about how multispecies communities differentially affect the host immune response compared to monomicrobial infections (Peters and Noverr, 2013). *In vitro,* mixed *C. albicans*-methicillin sensitive *S. aureus* (MSSA) biofilms contain secreted aspartyl proteases (SAP) and phospholipase C (PL-C) (Zago et al., 2015). Moreover, soluble factors produced in mixed *C. albicans*-MSSA biofilms are more damaging to epithelial cells compared to the factors produced in the respective single species biofilms (De Carvalho Dias et al., 2017). *In vivo*, *C. albicans* and *S. aureus* exhibit distinct immunomodulatory properties (Seidl et al., 2012), and during co-infection, an altered host immune response is observed, compared to the response in mono-species infections (Lin et al., 2009). For example, in an intra-abdominal catheter biofilm infection in mice, significantly more neutrophils were recruited to the peritoneum 24 h after co-infection with *C. albicans* and *S. aureus* compared to the monomicrobial infections. These results indicate a fast response and mobilization of neutrophils during mixed infection (Rogiers et al., 2018). Nash et al. studied the early stages of both local and systemic inflammatory events during mixed *C. albicans*-*S. aureus* intra-abdominal infection (Nash et al., 2014). They found that the synergistic lethality induced by co-infection is associated with host factors, including elevated local cytokines (IL-6, TNF-α, and IL-1β) (Nash et al., 2014). In the murine peritonitis model, a unique subset of innate pro-inflammatory cytokines (interleukin-6, granulocyte colony-stimulating factor, keratinocyte chemoattractant, monocyte chemoattractant protein-1, and macrophage inflammatory protein-1α) was identified to significantly increase during *C. albicans*-*S. aureus* co-infection, which leads to an increased inflammatory infiltration to the peritoneum and other target organs, compared to the monomicrobial infection (Peters and Noverr, 2013). These five cytokines were increased in both the kidneys and spleen during co-infections and are all involved in the innate proinflammatory response, activated very shortly after pathogen exposure. Additionally, IL-6, G-CSF, and KC are important for recruitment of neutrophils to the site of infection (Peters and Noverr, 2013). Recently, a novel role for the host immune response was suggested in facilitating *C. albicans*-*S. aureus* infectious synergy. Using time-lapse microscopy, it was demonstrated that macrophages are attracted to the hyphae of *C. albicans* and that they selectively engulf *S. aureus* cells attached to these hyphae, mediating *S. aureus* dissemination *in vitro* (Allison et al., 2019). Nevertheless, there is still a lot to discover in how co-infections of *C. albicans* with *Staphylococcus* species affect the host immune response or whether the host immune response against one microorganism affects the interaction with the other microorganism. Since insight into host-pathogen interactions are of fundamental concern, this topic should be addressed in future research (Figure 1C; Morales and Hogan, 2010).

## Treatment Strategies

Finding an efficient treatment strategy for polymicrobial infections is a major challenge since traditional therapies most often only target individual causative agents within specific kingdoms (Brogden et al., 2005). Combination therapy is being studied as a possible alternative to overcome this challenge. It was demonstrated that anidulafungin and tigecycline act synergistically on *S. aureus* in mixed *C. albicans*-*S. aureus* intra-abdominal catheter biofilm infections in mice (Rogiers et al., 2018). Other potential drug sources are also being investigated for combination therapy against polymicrobial infections. An example of such is the diverse group of “essential oils.” It was shown that *in vitro*, a combination of fluconazole and mupirocin with clove oil is, respectively, 10 and 4 times more effective against mixed biofilms than fluconazole or mupirocin alone (Budzynska et al., 2017). Recently, other important components of mixed communities of fungi and bacteria, such as quorum sensing and the production of ECM in biofilms, are being explored as possible targets. The effect of quorum quenching enzymes against *C. albicans*-*S. epidermidis* biofilms was recently studied (Weiland-Brauer et al., 2019). Two promising quorum quenching enzymes (QQ-5 and QQ-7), identified through functional screening of metagenomic large insert libraries, were found able to inhibit *C. albicans* morphogenesis, leading to an impaired biofilm formation. Moreover, they both inhibited *S. epidermidis* biofilm formation, probably by repression of polysaccharide intracellular adhesin (PIA), which plays a key role in biofilm formation of *Staphylococcus* species. Such quorum quenching molecules are promising drugs to combat mixed species infections (Weiland-Brauer et al., 2019). Small amphiphilic peptides are another class of antimicrobial proteins explored for their activity against (poly)microbial biofilms (Gupta et al., 2019). Recently, valine-glycine dipeptide-derived cholic acid, or CAP3, was found to exhibit broad-spectrum antimicrobial activity against both single and mixed *C. albicans*-*S. aureus* biofilms *in vitro* and *in vivo.* Its effect was attributed to both biofilm structure disruption and membrane permeabilization (Gupta et al., 2019). Furthermore, the activity of guanylated polymethacrylates, a new class of antimicrobial agents, was assessed against polymicrobial *C. albicans*-*S. aureus* biofilms (Qu et al., 2016). Guanylated polymethacrylates are synthetic structural mimics of antimicrobial peptides. They were found to be effective against established polymicrobial biofilms and they outperformed multiple other antimicrobial drug combinations. Finally, the sterilization of catheters against the formation of polymicrobial biofilms is also under investigation. Treatment of catheters with 50% ethanol for 4 h appeared to be sufficient to kill and inhibit the growth of *C. albicans*-*S. aureus* mixed biofilms (Peters et al., 2013). Unfortunately, effective measures to deal with polymicrobial infections consisting of *C. albicans* with *Staphylococcus* species are still lacking.

## Conclusion

Mixed infections of *C. albicans* with *Staphylococcus* species are of great importance in clinical settings. The enhanced antimicrobial tolerance, increased virulence, and altered infection outcome due to the interaction of both species makes it even more challenging to find efficient treatment strategies.

Although our knowledge on polymicrobial infections has been greatly expanded recently, there are still questions which remain to be solved in the future. The effect of mixed species infections on the host immune response is still not clear and should be further addressed. In addition, since candidemia is increasingly caused by non-*albicans Candida* species, it is interesting to study the interaction of *Staphylococcus* species with other *Candida* species such as *C. glabrata*, *C. auris*, *C. parapsilosis*, and *C. tropicalis*. Since our current knowledge on biofilms is still predominantly based on studies concerning monomicrobial or dual species biofilms, it would be interesting to include even more than two microorganisms. An environment where this is particularly important is within the oral cavity since it has a rich and diverse microbial flora. Understanding the complex interspecies communication and the microbial behavior when two or more species live together could help to improve our knowledge on polymicrobial infections and to develop effective treatment strategies.

## Author Contributions

HC and KD contributed equally to this publication. All authors contributed to the writing of this manuscript.

### Conflict of Interest Statement

The authors declare that the research was conducted in the absence of any commercial or financial relationships that could be construed as a potential conflict of interest.
